# Electrical Impedance Tomography Analysis Between Two Similar Respiratory System Compliance During Decremetal PEEP Titration in ARDS Patients

**DOI:** 10.1007/s40846-021-00668-2

**Published:** 2021-11-16

**Authors:** Po-Lan Su, Wei-Chieh Lin, Yen-Fen Ko, Kuo-Sung Cheng, Chang-Wen Chen

**Affiliations:** 1 Institute of Biomedical Engineering, National Cheng Kung University, Tainan, Taiwan; 2 Department of Internal Medicine, National Cheng Kung University Hospital, College of Medicine, National Cheng-Kung University, Tainan, 70403 Taiwan; 3 Medical Device Innovation Center, National Cheng Kung University, Tainan, Taiwan

**Keywords:** Positive end-expiratory pressure, Acute respiratory distress syndrome, Respiratory system compliance, Electrial impedance tomography

## Abstract

**Purpose:**

The positive end-expiratory pressure (PEEP) level with best respiratory system compliance (Crs) is frequently used for PEEP selection in acute respiratory distress syndrome (ARDS) patients. On occasion, two similar best Crs (where the difference between the Crs of two PEEP levels is < 1 ml/cm H_2_O) may be identified during decremental PEEP titration. Selecting PEEP under such conditions is challenging. The aim of this study was to provide supplementary rationale for PEEP selection by assessing the global and regional ventilation distributions between two PEEP levels in this situation.

**Methods:**

Eight ARDS cases with similar best Crs at two different PEEP levels were analyzed using examination-specific electrical impedance tomography (EIT) measures and airway stress index (SIaw). Five Crs were measured at PEEP values of 25 cm H_2_O (PEEP_25_), 20 cm H_2_O (PEEP_20_), 15 cm H_2_O (PEEP_H_), 11 cm H_2_O (PEEP_I_), and 7 cm H_2_O (PEEP_L_). The higher PEEP value of the two PEEPs with similar best Crs was designated as PEEP_upper_, while the lower designated as PEEP_lower_.

**Results:**

PEEP_H_ and PEEP_I_ shared the best Crs in two cases, while similar Crs was found at PEEP_I_ and PEEP_L_ in the remaining six cases. SIaw was higher with PEEP_upper_ as compared to PEEP_lower_ (1.06 ± 0.10 versus 0.99 ± 0.09, p = 0.05). Proportion of lung hyperdistension was significantly higher with PEEP_upper_ than PEEP_lower_ (7.0 ± 5.1% versus 0.3 ± 0.5%, p = 0.0002). In contrast, proportion of recruitable lung collapse was higher with PEEP_lower_ than PEEP_upper_ (18.6 ± 4.4% versus 5.9 ± 3.7%, p < 0.0001). Cyclic alveolar collapse and reopening during tidal breathing was higher at PEEP_lower_ than PEEP_upper_ (34.4 ± 19.3% versus 16.0 ± 9.1%, p = 0.046). The intratidal gas distribution (ITV) index was also significantly higher at PEEP_lower_ than PEEP_upper_ (2.6 ± 1.3 versus 1.8 ± 0.7, p = 0.042).

**Conclusions:**

PEEP_upper_ is a rational selection in ARDS cases with two similar best Crs. EIT provides additional information for the selection of PEEP in such circumstances.

**Supplementary Information:**

The online version contains supplementary material available at 10.1007/s40846-021-00668-2.

## Background

Tidal volume and positive end-expiratory pressure (PEEP) are two cardinal parameters in ventilator therapy of acute respiratory distress syndrome (ARDS) patients. Though the use of low tidal volume is well established, determining the optimal PEEP for selection remains challenging. A few available indicators are useful for selecting PEEP [1, 2], with the best respiratory system compliance (Crs) being a popular option [3]. The best Crs can usually be selected during the PEEP titration process, with or without recruitment maneuvers [3–5]. Similar best Crs (where the difference between the Crs of two PEEP levels < 1 cm H_2_O) can be identified at several PEEP levels sometimes. Selecting PEEP under such circumstances is challenging [3, 4]. A higher PEEP with addition of 2 cm H_2_O was adopted in the Alveolar Recruitment for Acute Respiratory Distress Syndrome Trial (ART) trial [3], while another trial selected a lower PEEP [4] but indicated no clear reasoning behind this selection. Electrical impedance tomography (EIT) is a noninvasive imaging technique that has the potential to provide new information for the ventilator management of ARDS patients [6]. Several examination-specific EIT measures have been developed to estimate the collapse/hyperdistension or recruitment/cyclic alveolar collapse proportion [7–9]. These EIT measures, which have been successfully applied to several animal and human studies, could facilitate the optimal selection of PEEP [5, 8, 10–12].

EIT has been employed at our hospital for PEEP choice in selected ARDS patients since 2014 and we have published a brief article discussing the issue of best PEEP level and recruitable lung volume [13]. We have also identified a few patients with two similar best Crs during decremental PEEP titration within the same study population. The aim of this study was to apply examination-specific EIT measures for regional ventilation analysis in ARDS patients with two similar Crs but different PEEP levels. In this study, the airway stress index (SIaw) [14, 15] was also calculated based on the shape of the airway pressure curve as a constant flow was used, and the lung volume was measured using the nitrogen washin-washout (NWI-WO) technique [16]. Our objective was to enable a rational selection of PEEP using examination-specific EIT measures and SIaw.

## Methods

### Study Population

In this study, ventilated patients over 18 years who fulfilled the diagnostic criteria of ARDS with a FiO_2_ requirement of ≥ 50% at our intensive care unit and PEEP > 8 cm H_2_O were screened for suitability between October 2014 and Febuary 2016. The exclusion criteria included (1) patients with metallic materials in the body (including wires, pins or implanted electrical devices); (2) patients with cutaneous diseases which prohibited the application of electrode leads to the body; (3) severe chronic obstructive pulmonary diseases or idiopathic pulmonary fibrosis; (4) hemodynamically unstable; (5) proved barotrauma (including pneumothorax or pneumomediastinum or subcutaneous emphysema); (6) pregnancy; (7) terminal malignancy or evidently irreversible diseases; (8) the use of extracorporeal membrane oxygenation (ECMO); (9) patients or family members who refused to participate in the study. The ethics committee of our hospital had approved this study (NCKUH-10403009). All patients who fulfilled the diagnostic criteria of ARDS received standard low tidal volume ventilator therapy (6–8 ml/Kg ideal body weight).

### Instrument and Measurement

Air flow and airway pressure were measured using a pneumotachograph (PN 155,362, Hamilton Medical, Bonaduz, Switzerland) and differential pressure transducers (P/N 113,252, Model 1110A, Hans Rudolph, Shawnee, KS, USA), respectively. The flow sensor was positioned between the endotracheal tube and Y-piece of the ventilator. Tidal volume was calculated by integrating the flow signal. All signals were sampled and digitized at 100 Hz, and the data were stored in a data-acquisition system (AcqKnowledge, Biopac MP150, Goleta, CA, USA). End-expiratory lung volume (EELV) was measured using the NWI-WO technique available via the GE Carestation ventilator (GE Healthcare, Chicago, Ill, USA) [16]. The airway plateau pressure 1 s after airway occlusion was denoted by P_pl_. PEEPt represented the total PEEP obtained with end-expiratory airway occlusion. We calculated △P = P_pl_ − PEEPt, while Crs was calculated to equal the tidal volume (Vt)/△P.

In this study, we employed a commercial EIT monitor (PulmoVista 500, Dräger Medical GmbH, Lubeck, Germany). PulmoVista 500 displays functional EIT images (i.e. relative impedance changes), which includes measurements of the tidal ventilation and changes in the end-expiratory lung impedance (EELI). The EIT data was registered at 20 Hz, low-pass filtered (35 per minute), and stored for offline analysis during the study.

### Lung Recruitment Protocol

We standardized the lung volume history using the extended sigh method for alveolar recruitment [17] prior to performing the lung recruitment assessments. PEEP was sequentially increased from baseline to 15, 20, and 25 cm H_2_O (every 30 s, from the baseline PEEP to a PEEP level of 25 cm H_2_O, twice). Vt was reduced by 25% from the previous baseline Vt during the incremental phase, while it was increased by 25% during the decremental phase. The upper limit of the peak airway pressure during this recruitment maneuver was 50 cm H_2_O. P_pl_ was determined at a PEEP of 25 cm H_2_O (PEEP_25_) and 20 cm H_2_O (PEEP_20_) during the second recruitment maneuver and following EELV determination using the NWI-WO method at a PEEP_H_, PEEP_I_, and PEEP_L_ of 15 cm H_2_O, 11 cm H_2_O, and 7 cm H_2_O, respectively. The recruited lung volume (Vrec) was calculated as the difference between the EELVs at PEEP_H_ and PEEP_L_ or PEEP_I_ and PEEP_L_, after subtracting the minimal deformable lung volume that was obtained by multiplying the Crs at PEEP_L_ with the PEEP difference [18]. The arterial blood gas was determined at the end of PEEP_H_, PEEP_I_, and PEEP_L_, with the EIT images simultaneously recorded.

### Stress Index Calculations from the Airway Pressure–Time Curve Profile Under Constant Flow

The equation used to fit the airway pressure–time (Paw-t) curve is given by airway pressure (Paw) = *a* * time (second)^*b*^ + *c*, where coefficient *a* represents the slope of the Paw-t relationship, and the coefficient *c* is the value of Paw at beginning (time_0_) and dimentionless coefficient *b* (SIaw) depicts the shape of the Paw-t curve. These coefficients were obtained using the Levenberg–Marquardt algorithm [15]. The shape of the Paw-t curve could indicate the tidal recruitment and hyperinflation. Ten tidal breaths were averaged and only the constant flow section was selected to ensure a good flow and airway pressure signal. We added 50 ms offsets at both ends of the constant flow section to further reduce its width [14, 15]. The above-detailed equation was also used to fit the selected time interval of the Paw-t curve. The three calculated SIaws were averaged at each PEEP level.

### Proportion of Recruitable Lung Collapse and Hyperdistension at Different PEEPs

The method proposed by Costa et al. [7] was used to calculate the degree of recruitable lung collapse and hyperdistension during decremental PEEP titration. The individual pixel impedance variations (△Z) between P_pl_ and PEEPt were computed. Pixel impedance compliance was computed as △Z/△P. The impedance compliance at five PEEP levels was determined for each pixel, and the amount of collapse or hyperdistension in the individual pixels was summed to estimate the corresponding percentages. No collapse or hyperdistension are observed at the highest and lowest PEEP levels, respectively.

### Cyclic Alveolar Collapse and Reopening During Tidal Breathing at Different PEEPs

The method proposed by Liu et al. [8] was used to estimate the cyclic alveolar collapse and reopening during tidal breathing at various PEEP levels. The lung regions were identified first, which at end-expiration included all pixel values > 25% of the maximum in the image. The lung regions corresponding to tidal breathing included all pixel values > 20% of the maximum in the tidal image. Regions ventilated during tidal breathing but not at end-expiration were associated with cyclic alveolar collapse and reopening. The degree of cyclic alveolar collapse and reopening was expressed in percentage values, which were calculated by dividing the absolute number of pixels associated with cyclic alveolar collapse and reopening by the total number of lung pixels during tidal breathing.

### Heterogeneity of Regional Lung Ventilation Distribution During Inspiration Using Intratidal Gas Distribution (ITV)

The method developed by Löwhagen et al. [9] was used to estimate the ITV. The inspiratory portion of the global tidal curve was divided into eight isovolumetric sections to calculate the ITV. The volume signal was first resampled and the isovolume points were calculated. Interpolation was used to obtain the corresponding EIT signals, which were divided into the nondependent (nondep) and dependent (dep) parts. The ratios of Vt_nondep_/Vt_dep_ in the eight equal volume parts were subsequently averaged to obtain the ITV index [5, 10, 19]. An ITV index of one indicated an equal regional ventilation distribution. An ITV index of less than one may indicate overdistension. A flow chart describing the steps used in ITV calculation could be found in the supplement material.

### Statistical Analysis

Data are presented as mean ± SD. Friedman’s analysis of variance for repeated measures was used to compare the ∆P, Vt, EELV, and arterial blood gas at the PEEP_H_, PEEP_I_, and PEEP_L_ levels. The independent samples t-test was used to compare two groups of normally distributed variables, while the Mann–Whitney U test was used for variables with non-normal distributions. All tests were two-sided, and a *p* value < 0.05 was considered statistically significant. All analyses were performed using Prism software, version 5 (GraphPad Software, San Diego, CA, USA).

## Results

### Study Population

During the study period, fifty-six cases were screened and 25 patients who met the Berlin’s criteria of ARDS entered our study. The male to female ratio is 20/5 and their mean age is 61.1 ± 16.3 years. Of the 25 patients receiving EIT and EELV measurement, two cases were terminated earlier because the measured lung volume was paradoxically higher under lower PEEP. Among the 23 patients who met the Berlin’s criteria of ARDS received EIT and EELV measurement following our standarad recruitment protocol. Two similar Crs was found during decremental PEEP titration in 8 patients. The hospital mortality rate of these 8 patients was 25%. Patients’ characteristics, outcomes and respective Crs at PEEP_H_, PEEP_I_, PEEP_L_ levels are shown in Table 1. Respiratory parameters over 5 PEEP levels, measured EELV, arterial blood gases and Vrec are shown in Table 2. For the two PEEP levels with similar Crs, the higher PEEP was designated as PEEP_upper_, the lower PEEP was designated as PEEP_lower_.

**Table 1:** Patients’ characteristics, outcomes and respective Crs at PEEP_H_, PEEP_I_, PEEP_L_ levels

Case	1	2	3	4	5	6	7	8
Gender/Age	M/65	F/40	M/30	M/73	F/64	F/67	M/36	M/41
MV day	11	2	6	3	3	3	3	13
ARDS Severity	Moderate	Moderate	Moderate	Moderate	Moderate	Moderate	Moderate	Moderate
Diagnosis	Pneumonia	Pneumonia, Turner syndrome	PJP, Behçet's disease	Pneumonia	Pneumonia	Pneumonia	Pneumonia	Pneumonia
Outcome	Survival	Survival	Survival	Death	Death	Survival	Survival	Survival
PEEP_H_ (cmH_2_O)	**15.6**	**14.7**	**14.5**	**14.3**	**13.9**	**14.8**	**14.6**	**15.4**
PEEP_I_ (cmH_2_O)	**11.4**	**10.4**	**10.3**	**10.7**	**9.4**	**10.6**	**10.8**	**11.6**
PEEP_L_ (cmH_2_O)	**6.8**	**6.2**	**6.1**	**6.6**	**5.2**	**6.5**	**6.8**	**7.7**
Crs, PEEP_H_ (ml/cmH_2_O)	**25.5**	**27.2**	**36.8**	**33.8**	**34.5**	**21.6**	**56.9**	**43.9**
Crs, PEEP_I_ (ml/cmH_2_O)	**32.8**	**31.8**	**36.1**	**37.5**	**34.6**	**27.9**	**59.0**	**54.3**
Crs, PEEP_L_ (ml/cmH_2_O)	**32.8**	**31.6**	**33.4**	**38.0**	**29.0**	**28.8**	**58.9**	**54.6**

*Crs* static respiratory system compliance, *F* female, *M* male, *MV* mechanical ventilation, *PEEP* positive end-expiratory pressure, *PEEP*_*H*_ PEEP level of 15 cmH_2_O, *PEEP*_*I*_ PEEP level of 11 cmH_2_O, *PEEP*_*L*_ PEEP level of 7 cmH_2_O, *PJP* pneumocystis jiroveci pneumonia

**Table 2:** Respiratory mechanics and arterial blood gas, EELV and Vrec

	PEEP_25_	PEEP_20_	PEEP_H_	PEEP_I_	PEEP_L_
PEEP (cmH_2_O)	24.2 ± 0.8	19.6 ± 0.7	14.7 ± 0.6	10.6 ± 0.7	6.5 ± 0.7
P_pl_ (cmH_2_O)	33.8 ± 3.6	32.0 ± 4.2	27.5 ± 3.6	21.7 ± 2.3	17.8 ± 2.0
△P (cmH_2_O)	9.7 ± 3.5	12.4 ± 3.9	12.7 ± 3.3	11.0 ± 2.2	11.3 ± 2.1
Vt (ml)	197.4 ± 54.7	317.8 ± 53.0	420.0 ± 70.8	416.6 ± 69.9	418.4 ± 72.0
C_rs_ (ml/cmH_2_O)	21.5 ± 6.1	28.1 ± 9.6	35.0 ± 11.3	39.3 ± 11.2	38.4 ± 11.8
pH	NA	NA	7.25 ± 0.08	7.26 ± 0.08	7.28 ± 0.08
PaCO_2_ (mmHg)	NA	NA	58.8 ± 11.2	57.6 ± 10.5	54.6 ± 8.8
PaO_2_/FiO_2_	NA	NA	188.6 ± 43.1*	164.0 ± 22.3	138.7 ± 16.2
EELV (ml)	NA	NA	1686.0 ± 452.1*	1504.0 ± 433.8	1146.0 ± 350.8
Vrec (ml)	NA	NA	227.6 ± 134.9	200.7 ± 101.3	

*△P* driving pressure, Ppl-PEEPt, *△Z* impedance variations, *ARDS* acute respiratory distress syndrome, *Crs* static respiratory system compliance, *EELV* end-expiratory lung volume, *ITV* intratidal volume distribution, *MV* mechanical ventilation, *nondep* non-dependent, *NWI-WO* nitrogen washin-washout, *Paw-t* airway pressure–time, *PEEP* positive end-expiratory pressure, *PEEP*_*25*_ PEEP level of 25 cmH2O, *PEEP*_*20*_ PEEP level of 20 cmH2O, *PEEP*_*H*_ PEEP level of 15 cmH2O, *PEEP*_*I*_ PEEP level of 11 cmH2O, *PEEP*_*L*_ PEEP level of 7 cmH2O, *PEEP*_*lower*_ The lower PEEP value of the two PEEPs with similar best Crs, *PEEP*_*upper*_ The higher PEEP value of the two PEEPs with similar best Crs, *Ppl* airway plateau pressure, *PEEPt* total PEEP, *SIaw* airway stress index, *Vrec* recruited lung volume, *Vt* tidal volume, *NA* not assessed

*p < 0.05 compared with PEEP_H_, PEEP_I_ and PEEP_L_

### Airway Stress Index (SIaw) Between PEEP_upper_ and PEEP_lower_

**Fig. 1:**
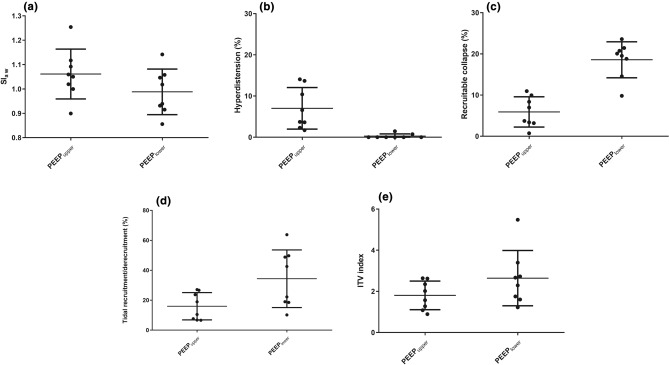
Airway stress index and examination-specific EIT measures between two PEEPs with similar best Crs. SIaw: airway stress index. ITV index: intratidal gas distribution index. PEEP_upper_: higher one of the two similar PEEPs. PEEP_lower_: lower one of the two similar PEEPs. **a** p = 0.05 **b** p = 0.0002 **c** p < 0.0001 **d** p = 0.046 **e** p = 0.042. *△P* driving pressure, Ppl-PEEPt. The difference between airway plateau pressure and total PEEP, *△Z* impedance variations. Impedance change during tidal ventilation, *Crs* static respiratory system compliance, *EELV* end-expiratory lung volume. Lung volume measured using nitrogen washin-washout method, *ITV* intratidal volume distribution. Quantitative measure of ventilation distribution using electrial impedance tomography, *NWI-WO* nitrogen washin-washout. An auxillary function for lung volume measurement in GE Carestation ventilator, Paw-t curve: airway pressure–time curve recorded at airway opeing, *PEEP*_*25*_ Actual total PEEP level with ventilator PEEP set at 25 cmH_2_O, *PEEP*_*20*_ Actual total PEEP level with ventilator PEEP set at 20 cmH_2_O, *PEEP*_*H*_ Actual total PEEP level with ventilator PEEP set at 15 cmH_2_O, *PEEP*_*I*_ Actual total PEEP level with ventilator PEEP set at 11 cmH_2_O, *PEEP*_*L*_ Actual total PEEP level with ventilator PEEP set at 7 cmH_2_O, *PEEP*_*lower*_ The lower PEEP in cases with two PEEPs of similar best Crs, *PEEP*_*upper*_ The higher PEEP in cases with two PEEPs of similar best Crs, *SIaw* Airway stress index. Namely the dimensionless coefficient *b* in airway pressure–time curve equation, (Paw) = *a* * time (second)^*b*^ + *c*, *Vrec* recruited lung volume. Calculated as the difference between the EELVs at PEEP_H_ and PEEP_L_ or PEEP_I_ and PEEP_L_, after subtracting the minimal deformable lung volume that was obtained by multiplying the Crs at PEEP_L_ with the PEEP difference

For PEEP_upper_, the SIaw ranged from 0.90 to 1.25 and SIaw was higher than 1.10 in two cases. For PEEP_lower,_ the SIaw ranged from 0.86 to 1.14 and SIaw was higher than 1.10 in one case and lower than 0.90 in one case. The SIaw of PEEP_upper_ was relatively higher than that of PEEP_lower_ (Fig. 1a).

### Recruitable Lung Collapse and Hyperdistension

Considering the PEEP_upper_ and PEEP_lower_ levels with similar Crs, PEEP_lower_ was associated with minimal hyperdistension (0%–1.5%), while PEEP_upper_ was associated with significantly higher hyperdistension (1.7%–14.1%). In contrast, PEEP_lower_ and PEEP_upper_ were associated with a higher (9.8%–23.6%) and much lower recruitable lung collapse (0.7%–11.0%), respectively (Fig. 1b, c).

### Tidal Recruitment/Derecruitment Between PEEP_upper_ and PEEP_lower_

Tidal recruitment/decruitment was associated with both PEEP_upper_ and PEEP_lower_, which ranged from 10.2% to 63.8% for PEEP_lower_ and 6.6% to 27.1% for PEEP_upper_. A significantly higher tidal recruitment/derecruitment was associated with PEEP_lower_ as compared to PEEP_upper_ (Fig. 1d).

### Intratidal Gas Distribution (ITV) Index Between PEEP_upper_ and PEEP_lower_

ITV index ranged from 1.2 to 5.5 in PEEP_lower_ and 0.9 to 2.6 in PEEP_upper_ (Fig. 1e). The ITV index was significantly higher with PEEP_lower_, implicating more heterogeneous ventilation distribution. The ITV index of PEEP_upper_ was less than 1 in one case, suggesting potential overdistention occurred if PEEP_upper_ + 2 cmH_2_O was selected. The PEEP_upper_ in this case was 14.5 cmH_2_O.

## Discussion

In this study, the two best Crs had significantly different ventilation distributions, under similar ventilator settings and different PEEP levels. The main findings were as follows: (1) A significantly higher proportion of recruitable collapse and tidal recruit-derecruit were linked to PEEP_lower_, while PEEP_upper_ was associated with a higher proportion of hyperdistension. (2) PEEP_upper_ might be a more appropriate selection when considering ventilation homogeneity and recruitable collapse. However, lung overdistension may be an issue in case when PEEP_upper_ is in PEEP_H_ range. The use of examination-specific EIT measures in these patients provided important information which may allow personalized choice of PEEP in ARDS patients.

The choice of the PEEP level in ARDS patients has always been debated. In recent years, individualized titration has been the preferred method due to the heterogeneity observed in ARDS patients [1]. A wide variation in the ventilation distribution was observed in our patients despite PEEP selection based on the best Crs, which is consistent with the current viewpoint. SIaw, which describes the time course of the airway pressure profile under constant flow conditions, is an established parameter for the appropriate selection of PEEP in ARDS patients [14]. SIaw > 1.10 and SIaw < 0.90 indicated tendencies towards lung hyperdistension and collapse, respectively [15]. SIaw tends to be higher in PEEP_upper_ and lower in PEEP_lower_. SIaw was not observed in the recommended range (0.90 < SIaw < 1.10) in two PEEP_upper_ cases and two PEEP_lower_ cases. Thus, this indicates that either best Crs did not always ensure a safe SIaw.

Recruitable lung collapse and lung hyperdistension are two undesirable conditions that may cause lung injury [20]. The method proposed by Costa et al. [7] was used to quantitatively evaluate the above-mentioned conditions. Selecting the best PEEP level with minimal lung collapse and hyperdistension is challenging due to their concomitant presence in the lung. A collapse level of up to 10–15% is an acceptable safety margin with minimal hyperdistension, which is the more undesirable condition [7, 12]. In this study, we found that 7 cases had a recruitable lung collapse above 10% and 6 cases had a recruitable lung collapse above 15% when PEEP_lower_ was selected. In contrast, only 1 case had a recruitable lung collapse above 10% and none above 15% when PEEP_upper_ was selected. The tidal recruited/derecruited percentage, which was calculated using the method proposed by Liu et al. [8], was significantly higher at PEEP_lower_. Thus, the present evidence from the EIT analysis suggests that the selection of PEEP_upper_ may be more appropriate when considering the level of recruitable alveolar collapse. However, lung hyperdistension remains a concern, as it is understandably higher with PEEP_upper_. Though lung hyperdistension is minimal with PEEP_lower_, 3 of our cases would have an EIT-derived hyperdistension greater than 10% with PEEP_upper_. The level of lung hyperdistension obtained from EIT has been known to overestimate the actual hyperdistension from the CT images [7]. Thus, we additionally used the ITV index to determine the appropriate PEEP level. ITV index is an useful indicator of ventilation homogeneity. An ITV index of one indicates a homogeneous tidal volume distribution in the non-dependent and dependent lung regions. ITV index was higher at PEEP_lower_ than that at PEEP_upper_, suggesting better ventilation homogeneity with PEEP_upper_. However, ITV index of one patient was < 1 when PEEP_upper_ was selected, which implicated overdistention might have occurred when PEEP_upper_ + 2 cm H_2_O was applied.

Our study has several limitations. First, we investigated a small sample size of patients in this study. However, these are all ARDS patients and our physiological recordings combined with EIT analysis provided significant relevant information with respect to the two similar Crs levels. These information provided additional clues in the selection of PEEP. Second, we used a limited pressure range for the recruitment maneuvers. A small fraction of lung recruitment might require higher pressures to open [21]. The EIT analysis might have differed for different recruitment maneuvers. Third, we only employed EIT and physiological measurements and did not perform a chest CT scan, which is a gold standard for assessing the collapsed and recruitable lung tissue. Furthermore, EIT measures were obtained only for a portion of the lung region. However, the reliability of EIT analysis techniques has been confirmed [6] and the results of present study were in good agreement with physiological reasoning. EIT provides valuable information on the regional ventilation, which could potentially aid our decisions in ventilator therapy [22].

In conclusion, although PEEP_upper_ is preferred for ARDS patients with two similar best Crs but different PEEP levels from our EIT study, the use of EIT clearly revealed the heterogeneous ventilation distribution in individual ARDS patient under two similar best Crs. We recommend addition of examination-specific EIT measures in this difficult-to decision circumstances to select the most appropriate PEEP which should be of value in our ventilatory management of individual ARDS patient.

## Supplementary Information

Below is the link to the electronic supplementary material.

Supplementary file1 (docx 67 KB)
