# Supplementary material for: Electrical Impedance Tomography Analysis Between Two Similar Respiratory System Compliance During Decremetal PEEP Titration in ARDS Patients
Source: J Med Biol Eng. 2021 Nov 16;41(6):888–94. doi: 10.1007/s40846-021-00668-2 (PMC8593398; doi:10.1007/s40846-021-00668-2)

**Calculation of the intratidal gas distribution (ITV) index**

Intratidal gas distribution (ITV) is calculated during the inspiratory phase. The inspiratory tidal volume is divided into 8 equal volume segments and adapted to the duration of tidal EIT signal. The procedures are done in the following order:

1. Selection of the inspiratory tidal breath phase
2. Estimation of the inspiratory EIT duration
3. Resampling of the integrated volume sequence to fit the EIT signal sequence
4. Division of the integrated volume into 8 isovolume segments
5. Placement of the 8 isovolume segments into their neighboring locations in the resampled volume sequence. Calculation of the ratio of the difference in the EIT to the corresponding difference in the neighboring volume sequence (E/V ratio). Calculation of the volume difference between the value of the isovolume locus and its neighboring volume sequence and multiplication of the E/V ratio to obtain the value of the corresponding difference in impedance
6. The EIT signal could thus be constructed at the exact isovolume locus.
7. The ventral and dorsal ventilation distribution at each segment could be determined, and the ratio between ventral and dorsal ventilation could be calculated. The ITV index was an average of the 8 segments. A demonstration figure is shown below, and a demonstration file could be found as an Excel file.


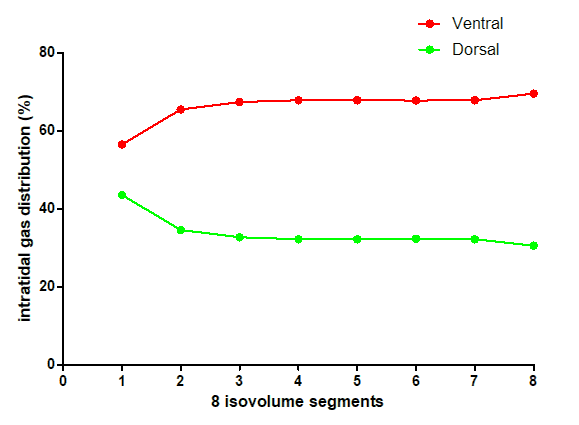

Supplement: Supplementary file 1 — Supplementary file1 (docx 67 KB) [file 40846_2021_668_MOESM1_ESM.docx]
